# Clinico-demographic and molecular characteristics of familial Mediterranean fever in Egypt: a multicenter study

**DOI:** 10.1038/s41598-026-55905-y

**Published:** 2026-07-25

**Authors:** Mohamed Elbadry, Noha H. Eltaweel, Mohamed Hussein Ahmed, Aya M. Mahros, Fathiya El-Raey, Shamardan Bazeed, Hanaa Omar, Ahmed Shamardan, Mahmoud Hagag, Shimaa Arafat, Asmaa Bakr, Marwa Tahoon, Sara M. Sayed, Samy Zaky

**Affiliations:** 1 Endemic Medicine Department, Capital University, 2 Ahmed Elzomor str., Cairo, Egypt; 2 Medical Molecular Genetics Department, National Research Centre, 33 Elbohoth str., Dokki, Giza, Egypt; 3 Hepatology, Gastroenterology and Infectious Diseases Department, Faculty of Medicine, Kafrelsheikh University, Kafr Elsheikh, Egypt; 4 Department of Hepato-gastroenterology and Infectious Diseases, Damietta faculty of Medicine, Al-Azhar University, Damietta, Egypt; 5 Department of Clinical Sciences, College of Medicine, Sulaiman Alrajhi University, Al Qassim, Saudi Arabia; 6 Tropical Medicine and Gastroenterology, Qena University, Qena, Egypt; 7 Department of Hepato-Gastroenterology and Infectious Diseases, Al-Azhar University, Cairo, Egypt; 8 Faculty of Medicine, National South Valley University, Qena, Egypt; 9 Gastroenterologist and Hepatologist, Ministry of Health, Cairo, Egypt; 10 Epidemiology and Preventive Medicine Department, National Liver Institute, Menoufia University, Menoufia, Egypt; 11 Biochemistry and Molecular Biology Department, Faculty of Pharmacy (Girls), Al-Azhar University, Cairo, Egypt

**Keywords:** Familial Mediterranean fever, *MEFV* gene, Clinico-demographic features, Molecular genetics, DNA, Diseases

## Abstract

Familial Mediterranean Fever (FMF) is an inherited autoinflammatory disease characterized by recurrent episodes of fever and serositis caused by mutations in the MEFV gene. FMF primarily affects individuals of Mediterranean ancestry. Typical manifestations include short-lasting attacks of abdominal pain, chest pain, and arthritis. Long-term complications, such as amyloidosis, might be prevented with colchicine treatment. This study aimed to assess the clinical, demographic and molecular features of FMF in Egyptian patients. This multicenter prospective study included 280 clinically suspected FMF patients referred to the outpatient clinics of participating centers. Patients were enrolled based on recurrent episodes of fever and abdominal pain suggestive of FMF. The diagnosis of FMF was established based on the Eurofever/PRINTO criteria, in addition to molecular genetic confirmation of MEFV mutations. Patients who did not meet diagnostic criteria were excluded. The study included 173 female patients, and 107 male patients with age range from 2 up to 60 years. All patients were descending from different families. Parental consanguinity was found in 24.6% while positive family history was seen in 33.2%. The duration of the attack in almost two third of the patients was less than 48 h, only 6% of the patients suffered attacks longer than 72 h. About half the patients achieved a marked reduction or complete cessation of FMF attacks, along with normalization or significant reduction of inflammatory markers (e.g., serum amyloid A), following colchicine therapy at a daily dose of 1.5–3 mg., only 6% needed higher doses. The initial serum amyloid A was normal in 47.5% in the patients and elevated in the remaining patients. Fever was documented in only 19.6% of patients at presentation; abdominal pain was among the most common presentation, seen in 62.1%. The most frequently observed MEFV allele was E148Q (143, 39.5%) followed by M694I (*n* = 59, 16.3%), A744S and V726A (*n* = 44, 12.2% for each), then M680I (*n* = 35, 9.7%). In our multicenter study focused on Egyptians, 93.6% of the enrolled FMF patients carried at least one MEFV variant. The E148Q allele was the most frequently observed followed by M694I. The study also revealed that Egyptian patients exhibited a mild form of the disease with a female predominance. This mild presentation might be attributed to the high E148Q prevalence and low rate of amyloidosis in our cohort.

## Introduction

The most prevalent inherited recurring fever syndrome is familial Mediterranean fever (FMF)^1^. Siegal published the first description of it as a novel entity in 1945 ^2^. Although FMF is now known in many regions of the world, it was first identified in patients from the Eastern Mediterranean region, specifically among Sephardic Jews, Armenians, Turks, and Arabs^3^. Indeed, migrations and increased diagnostic sensitivity are causing its epidemiology to change quickly. It is currently widespread, affecting an estimated 200,000 or more people globally^4^.

Although it usually starts in infancy or early adulthood, and it is commonly detected years after it first appears^5^. Additionally, this illness may manifest later in life, and adult patients are frequently undiagnosed. A number of things can cause delays in diagnosis. For instance, it is frequently misunderstood that individuals with FMF must be of Mediterranean heritage or that those with autoinflammatory diseases must have homozygous mutations in order to exhibit symptoms^6^.

The severity and frequency of FMF manifestations vary. Accurate diagnosis depends on recognizing symptom distribution during acute episodes^7^. The disease is marked by recurrent, self-limited fever attacks accompanied by serositis, arthritis, and abdominal pain; amyloid A amyloidosis is the main long-term complication^8^. Because both genetic and environmental factors influence expression, symptom frequency and intensity differ among patients. Secondary amyloidosis remains the leading cause of morbidity and mortality^4^.

FMF is a subtype of monogenic autoinflammatory diseases (AID), a diverse collection of medical disorders marked by innate immunity dysregulation occurs by mutations in genes related to inflammation regulation^9^.

Gain-of-function mutations in the Mediterranean fever gene (*MEFV*; OMIM: 608107), found on chromosome 16 (16p13.3) and contains 10 exons, are the cause of FMF. Pyrin, a 781 amino acid protein that has several isoforms in the cytoplasm and nucleus, is encoded by *MEFV*^10,11^.

However, the majority of patients seem to have an autosomal recessive inheritance pattern rather than a dominant one because the mutations have limited penetrance and the gene’s expression is dose dependent. However, in 30% of cases, the disease might be expressed with a single mutant allele^12^. Following the discovery of the *MEFV* gene, it was also shown that not all mutations in the gene result in an FMF phenotype of the same intensity. Particularly, the M694V mutation in exon 10 has been found to cause more severe symptoms, increased colchicine resistance, and FMF-related sequelae include sacroiliitis, arthritis, amyloidosis, and erysipelas-like erythema (EBE)^11^.

More than 180 gene polymorphisms have been identified in FMF affected patients. Mutations have been found especially in exons 2, 3, 5 and mostly in exon 10 of the MEFV gene. The four missense mutations (M6801, M694V, M694I and V726A) located on exon 10, in addition to E148Q mutation located on exon 2, are expected as the five founder mutations that account for the genotype of the vast majority of FMF patients^13^, with different frequencies in affected populations (Armenians, Arabs, Jews, and Turks)^14^. M694V homozygous mutation shows more severe clinical features than other mutations and had been reported with a relative resistance to colchicine^15^. It is worth mentioning that heterozygous mutation in the MEFV gene can also cause acute febrile neutrophilic dermatosis (AFND; OMIM: 608068), which shows some overlapping features.

Achievement of minimal or no clinical activity is the treatment goal in FMF. Complete control of ongoing subclinical inflammation to prevent complications is strongly recommended^10^. Colchicine is the standard first-line therapy of FMF and should start as soon as a clinical diagnosis is made. The patient’s adherence to treatment is crucial. Long-term colchicine prophylaxis is essential to control inflammation and prevent attacks of clinical manifestations as well as AA amyloidosis^16^. Interleukin − 1 (IL-1) inhibitors have the potential to improve patient outcome even in FMF patients with co-morbidities or severe complications in whom inflammation control is difficult to achieve with colchicine alone^17^.

Despite significant advances in our understanding of FMF, many aspects of the disease remain unclear. For example, the exact mechanisms underlying the development of FMF-associated amyloidosis are not fully understood. Additionally, there is a need for more effective treatments for patients with severe or refractory FMF^2^.

Therefore, this study aims to comprehensively describe the clinical, demographic, and molecular characteristics of Egyptian FMF patients, evaluate genotype–phenotype associations, and assess their response to colchicine therapy.

## Patients and methods

1. Patient selection and diagnostic criteria.

This multicenter prospective study included 280 clinically suspected FMF patients referred to the outpatient clinics of participating centers between January 2023 and December 2024. Parental and extended family testing was not systematically performed in this study due to logistical and resource limitations. Families were offered genetic counseling by the healthcare provider as part of the study protocol. Counseling included explanation of the genetic findings, inheritance patterns of Familial Mediterranean Fever (FMF), and recommendations for screening of at-risk relatives. However, uptake of counseling varied, and not all families were able to attend due to logistical constraints. During the study period, none of the patients requested prenatal testing, which may be attributable to the absence of a gynecologist within the multidisciplinary team.

2. The study cohort comprised patients diagnosed with FMF. Diagnosis was established based on the internationally recognized Eurofever/PRINTO classification criteria (2020)^1^. In accordance with these criteria, patients were included if they fulfilled one of the following genetic-clinical profiles: Profile A (Confirmed Genotype): Presence of two pathogenic MEFV variants and at least one supporting clinical criterion. Profile B (Clinical Diagnosis with Incomplete Genotype): Presence of a single pathogenic MEFV variant (heterozygous state) and fulfillment of at least two clinical criteria. All patients also exhibited a clinical phenotype consistent with the classic Tel-Hashomer criteria at enrollment, reinforcing the characteristic nature of their presentation. Genotypic analysis was successfully completed for 276 patients. For four patients, genetic data were unavailable due to technical sample issues. These four patients were retained in the cohort as they presented with a severe and highly characteristic clinical phenotype that fully satisfied the clinical components of both the Tel-Hashomer and Eurofever/PRINTO criteria. A sensitivity analysis confirmed that the exclusion of these four patients did not alter the study’s primary conclusions.

### Inclusion and exclusion criteria

Patients were recruited from Helwan University Hospital, Kafr Elsheikh University Hospital, Al-Zahraa University Hospital, Al-Azhar University Hospital for girls in Damietta, Qena Univerity Hospital between January 2023 and December 2024. The inclusion criteria were as follows:

- Inclusion Criteria:
  1. A clinical presentation of recurrent febrile episodes or serositis attacks (≥ 3 attacks) accompanied by symptoms such as abdominal pain, arthritis, or pleuritic chest pain.
  2. A confirmed diagnosis of Familial Mediterranean Fever (FMF) according to the Eurofever/PRINTO classification criteria (2020). This includes patients with:
- A confirmed genotype (two pathogenic MEFV variants) and at least one clinical criterion.
- A non-confirmed genotype (e.g., a single pathogenic MEFV variant) and at least two clinical criteria.
  3. Willingness and ability to provide written informed consent.
- Exclusion Criteria:
  1. Incomplete clinical records that precluded accurate classification.
  2. The identification of an alternative diagnosis that could explain the symptoms (e.g., active infection, malignancy, or another defined autoinflammatory syndrome).
  3. Loss to follow-up or non-compliance with the study protocol that prevented the collection of essential outcome data.

### Sample size

Based on previous literature, the estimated prevalence of FMF in Egypt is approximately 0.1%. To achieve sufficient power, the required sample size was calculated to be 154 participants at a 95% confidence level and 0.5% margin of error; however, 280 patients were ultimately enrolled to enhance representativeness.

### Data collection and laboratory investigations

Demographic and clinical data—including age, gender, ethnicity, family history, and attack characteristics (frequency, duration)—were retrieved from the Hospital Information Management System (HIS) and verified through structured interviews. A thorough physical examination assessed organomegaly, surgical scars, and signs of active inflammation.

Laboratory investigations were performed for two primary purposes:

1. **Characterization of Acute Attacks**: To confirm and assess the inflammatory burden during self-reported flare-ups, acute-phase reactants (APR) including C-reactive protein (CRP), erythrocyte sedimentation rate (ESR), and leukocyte count were measured during acute attacks.
2. **Assessment of Baseline Inflammation and Amyloidosis Risk**: To evaluate subclinical inflammation and long-term risk during clinically quiescent periods, key biomarkers were measured during attack-free remission phases. This included serial measurements of Serum Amyloid A (SAA), a critical marker for amyloidosis risk stratification.

Routine laboratory panels, including complete blood count (CBC), liver function tests (ALT, AST), and renal function tests (blood urea, serum creatinine), were performed periodically as part of standard care. Proteinuria was assessed via 24-hour urine protein quantification (mg/day).

Importantly, for the comparative analysis of clinical characteristics between patient genotypes (e.g., as presented in Table 4), the APR and SAA values used were those recorded during documented attack-free remission periods. This approach allows for a meaningful comparison of baseline inflammatory states and amyloidosis risk profiles among different genetic subgroups.

N.B.1: The management of patients in the present study was aligned with established treatment recommendations for FMF. Specifically, patients identified as homozygous for the p.Met694Val variant or compound heterozygotes carrying p.Met694Val and another pathogenic allele were maintained on lifelong colchicine therapy, administered at standard weight- and age-adjusted doses. For patients without the p.Met694Val variant and those with milder disease phenotypes (i.e., infrequent inflammatory attacks), management was individualized; these patients either received colchicine therapy or were closely monitored at regular intervals (every six months) for early detection of proteinuria and disease progression.

N.B.2: A favorable response was defined as complete remission (absence of FMF attacks) or a marked reduction in attack frequency (≥ 50% reduction) accompanied by normalization or a significant decline in inflammatory markers, particularly SAA, compared with baseline values. The time frame for response assessment was also clarified. Treatment response was evaluated over a minimum follow-up period of 6 months after initiation or dose adjustment of colchicine, with regular clinical and laboratory monitoring.

### Genetic testing

Blood samples were collected from all participants in EDTA tubes. Genomic DNA was isolated from 2 ml of peripheral blood using the QIAamp DNA Mini Kit (QIAGEN, Hilden, Germany). FMF targeted variant detection was carried out using the FMF Multiplex Real Time PCR Kit (Cat. no: 11R-20-20; SNP-Biotechnology, Ankara, Turkey). This kit analyzes twenty mutations, which are: E84K in exon 1, L110P, E148Q, E148V, E167D, E230K/Q, T267I, P283L and G304R in exon 2, P369S in exon 3, F479L in exon 5, and in exon 10 M680I (G/C-A), M694I, M694V, K695R, V726A, A744S, R761H. PCR amplification was carried out according to the manufacturer’s instructions for exons 1, 2, 3, 5, and 10. PCR program was set as (95 °C × 30 s; 30 cycles of 55 °C × 30 s and 72 °C × 30 s). Genetic results were obtained for 276 out of the 280 enrolled patients.

### Ethical considerations

This study was conducted in strict accordance with international ethical guidelines, having received formal approval from the Helwan University Faculty of Medicine Ethics Committee (Approval No.: 139–2024). Prior to participation, written informed consent was obtained from all adult participants or legal guardians of minor participants after comprehensive explanation of the study objectives and procedures. The research adhered rigorously to the ethical principles outlined in the Declaration of Helsinki and Good Clinical Practice (GCP) guidelines, ensuring the protection of participants’ rights, confidentiality, and welfare throughout all stages of the investigation. These measures were implemented to maintain the highest standards of ethical research conduct while collecting and analyzing sensitive clinical and genetic data.

### Statistical analyses

All statistical calculations were performed using the SPSS 22 (IBM SPSS Statistics for Windows, Version 22.0. NY: IBM Corp). Statistical significance for all analyses was accepted at *p* < 0.05. Categorical values were expressed as percentages, frequency and non-categorical variables were given as the mean ± standard deviation (SD). Chi-square tests were used to analyze categorical data. Student *t*-test and MannWhitney U test were used to analyze continuous data.

## Results

### Patient demographics

**Fig. 1:**
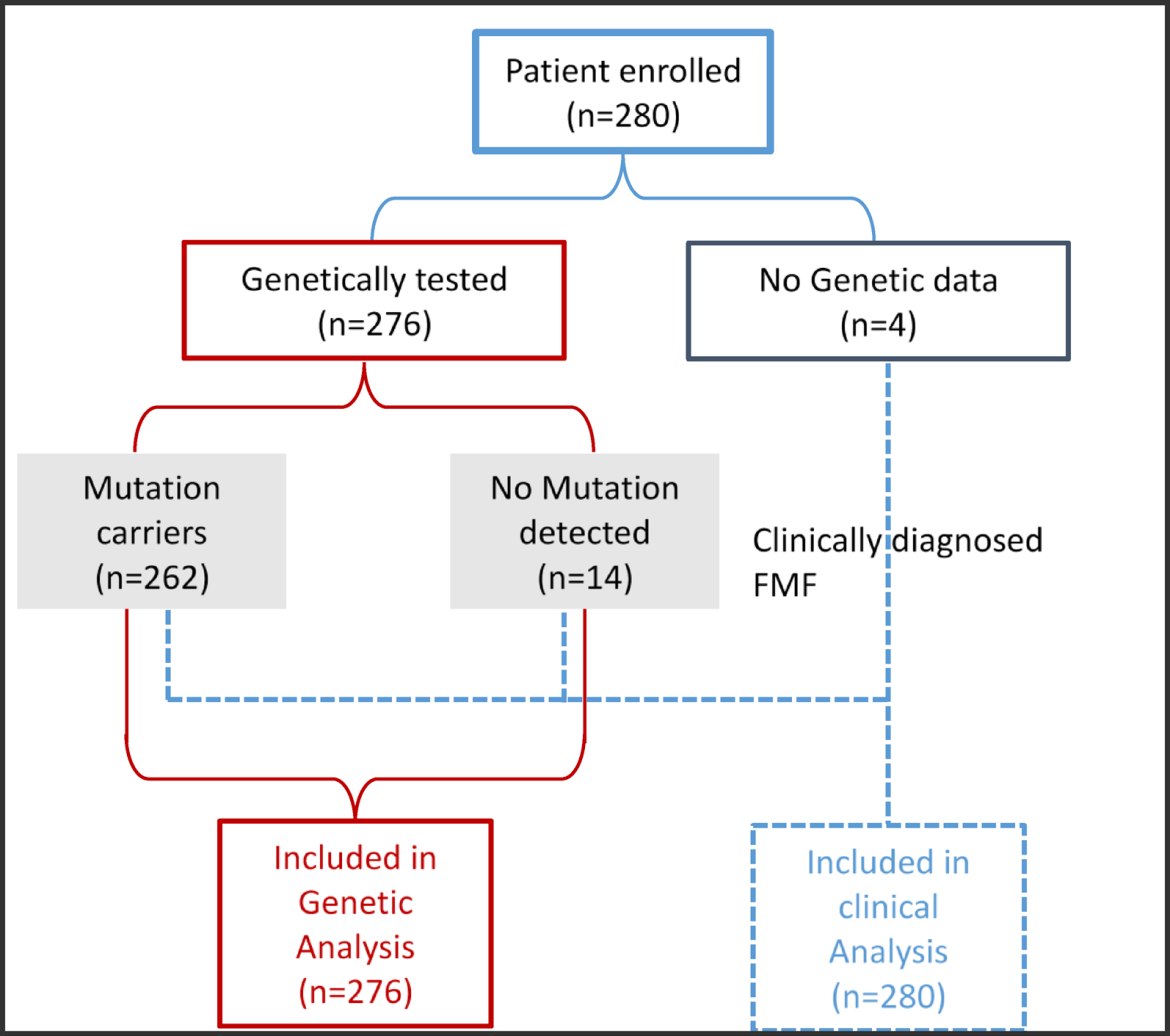
Flowchart illustrating patient enrollment, genetic testing, and final cohort classification. Genetic analyses were performed on patients with available molecular data (*n* = 276), while clinical analyses included the entire cohort (*n* = 280), including patients diagnosed based on clinical criteria alone.

A total of 280 Egyptian patients (173; 62% were female, and 107 were male) were enrolled in this study. The demographic and clinical data of the patients are listed in (Table 1). The age of patients ranged from 2 up to 60 years with mean 22.6 ± 12.3 years. All patients were descending from unrelated families. Out of 280 patients, 109 (38.9%) were in pediatric group (< 18 years) and 171 (61%) were in adult (≥ 18 years) group. All participants were of Egyptians. Figure 1 shows a flowchart of the enrolled patients.

Almost two third of the patients had negative FMF family history. Parental consanguinity was found in 24.6% while positive family history was seen in 33.2%. The number of attacks per year was variable, such that 42% of the patients suffered > 6 attacks, 41% of the patients suffered 3–6 attacks, and 16.8% of the patients only suffered < 3 attacks per year, however data on the number of attacks per year were missing for 60 patients. The duration of the attack in almost two third of the patients was less than 48 h, only 6% of the patients suffered attacks longer than 72 h. History of laparotomy or abdominal surgery was found in 18.9% of the patients. The sonographic finding was normal in almost half the patients, while others showed splenomegaly, pelvic collection, colonic distention/ tenderness, fatty liver and renal gravies/cystitis in 22.9, 12.1, 8.6, 6.1 and 4.6% of the patients; respectively.

About half the patients suffered remission with colchicine dose ≤ 1.5 mg/day and a marked reduction or complete cessation of FMF attacks, along with normalization or significant reduction of inflammatory markers (e.g., serum amyloid A), following colchicine therapy at a daily dose of 1.5–3 mg., only 6% needed higher doses. None of the patients required anti-IL-1 therapy.

The initial serum amyloid A was normal in 47.5% of the patients and elevated in the remaining patients (*n* = 202). There was no clinical significance in gender groups in correlation with number of attacks, duration of attacks and response to colchicine treatment. However, 10 (3.6%) patients exhibited proteinuria > 300 mg/day.

**Table 1:** Demographic data and clinical data of the studied patients (*N* = 280).

Variables			(no = 280)
Age at time of diagnosis (years)	Range		2.0–60.0
	Mean ± SD		22.6 ± 12.3
Gender	Male		107 (38.2%)
	Female		173 (61.8%)
Residence	Rural		179 (63.9%)
	Urban		101 (36.1%)
Family history of FMF	Negative		187 (66.8%)
	Positive		93 (33.2%)
Parental consanguinity	Negative		211 (75.4%)
	Positive		69 (24.6%)
Delay in confirming diagnosis	No		35 (16.8%)
	< One year		59 (21.1%)
	≥One year		114 (40.7%)
Number of attack/year (missing = 60) *n* = 220	< 3		37 (16.8%)
	3–6		90 (40.9%)
	> 6		93 (42.2%)
Duration of every attack (missing = 52) *n* = 228	< 48 h		143 (62.7%)
	48–72 h		71 (31.1%)
	> 72 h		14 (6.1%)
History of laparotomy / abdominal surgery (yes)	53 (18.9%)		
Sonographic finding	Normal		128 (45.7%)
	Splenomegaly		64 (22.9%)
	Pelvic collection		34 (12.1%)
	Fatty liver		17 (6.1%)
	Colonic distension/tenderness		24 (8.6%)
	Renal gravies/cystitis		13 (4.6%)
Colchicine dose response [mg/day] (missing = 31) *n* = 249	< 1.5		116 (46.6%)
	1.5–3		118 (47.4%)
	> 3		15 (6%)
Initial serum Amyloid (*n* = 202)	Normal		96 (47.5%)
	Raised		106 (52.5%)
Level of Amyloid A (mg/L)	Median (Range)		24.0 (1.3–222.0)
	Mean ± SD		41.4 ± 50.2
Hemoglobin (g/dL)	Median (Range)		12.0 (8.1–16.1)
	Mean ± SD		11.2 ± 3.6
WBCs [×10^3^] (cell/mm^3^)	Median (Range)		7.6 (4.3–18.6)
	Mean ± SD		7.06 ± 3.2
PLTs [×10^3^] (cell/mm^3^)	Median (Range)		233.0 (132.0–515.0)
	Mean ± SD		253.2 ± 74.5
ESR (mm/h)	Median (Range)		27.0 (3.0–27.0)
	Mean ± SD		30.2 ± 16.8
CRP (mg/L)	Median (Range)		9.0 (5.0–233.0)
	Mean ± SD		14.3 ± 23.0
Proteinuria (n)	above 300 mg/day	10 (3.6%)	

### Clinical presentation of FMF patients and sonographic findings

**Fig. 2:**
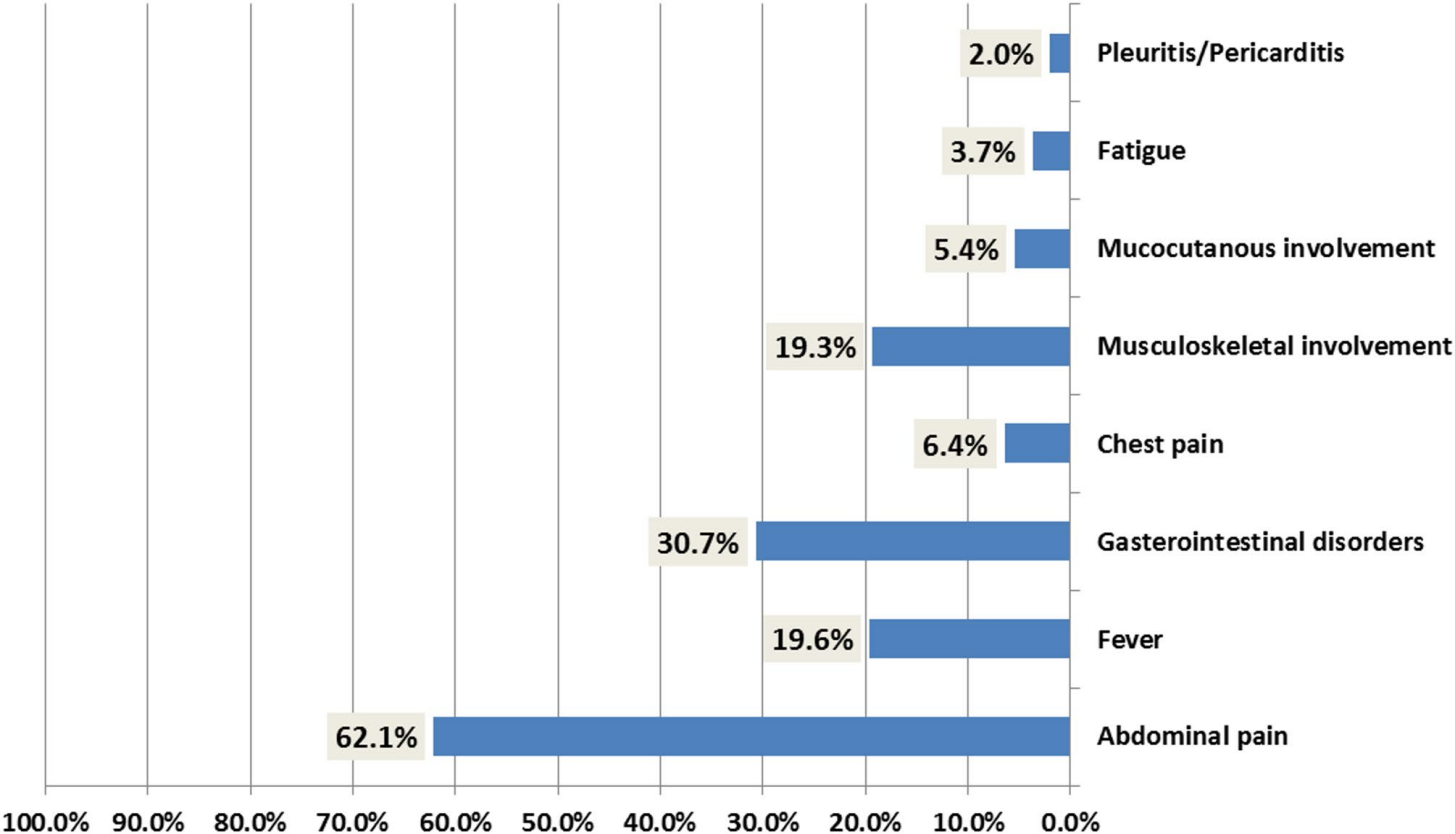
Clinical presentation of FMF patients.

While fever is a namesake feature of FMF, it was documented in only 19.6% of patients at presentation. This aligns with modern clinical series describing a high frequency of incomplete or atypical attacks, where localized inflammatory symptoms (e.g., abdominal or musculoskeletal pain) occur without prominent fever; abdominal pain was among the most common presentation, seen in 62.1%, followed by gastrointestinal disorders (30.7%), musculoskeletal involvement (19.3%), chest pain (6.4%) and mucocutaneous involvement (5.4%) (other clinical manifestations are represented in Fig. 2). All patients had normal ocular findings.

### FMF genetics and relation to attacks

The MEFV genotype in our patients are presented in Table 2. MEFV mutations were found in 262 of the 280 patients (93.6%). The genotype of 276 patients was analyzed, while that of four patients was missed. Among those patients, 41 (14.9%) were homozygous, 59 (22.4%) were compound heterozygous while 162 (58.7%) were heterozygous, while 14 patients (5.1%) had no mutations according to the used test strip. The MEFV allele frequencies are shown in Table 3. The most frequently observed allele was E148Q (143, 39.5%) followed by M694I (*n* = 59, 16.3%), A744S and V726A (*n* = 44, 12.2% for each), then M680I (*n* = 35, 9.7%).

**Table 2:** The genotypes of the studied patients (*N* = 276).

Genotype	*n*	%
No mutation	**14**	**5.1%**
Homozygous	**41**	**14.9%**
E148Q	23	8.3%
A744S	6	2.2%
M680I	4	1.4%
M694I	3	1.1%
V726A	3	1.1%
M694V	2	0.7%
Heterozygous	**162**	**58.7%**
E148Q	63	22.8%
V726A	23	8.3%
M694I	22	8.0%
A744S	21	7.6%
M680I	18	6.5%
M694V	9	3.3%
R202Q	3	1.1%
P369S	2	0.7%
Compound heterozygous	**59**	**21.4%**
E148Q, M694I	13	4.7%
E148Q, A744S	11	4.0%
M6801 (G/C), M694I	9	3.3%
M694I, V726A	8	2.9%
E148Q, V726A	6	2.2%
M694V, R761H	6	2.2%
E148Q, I692del	2	0.7%
E148Q, L110P	1	0.4%
E148Q, P369S	1	0.4%
M694V, M694I	1	0.4%
M694V, V726A	1	0.4%
Total	**276**	**100.0%**

**Table 3:** The MEFV allele frequency in our patients.

#	Allele	HGVS nomenclature	no	%
1	E148Q	**NM_000243.2(MEFV): c.442G > C**	143	39.5%
2	M694I	**NM_000243.2(MEFV): c.2082G > A**	59	16.3%
3	A744S	NM_000243.2(MEFV): c.2230G > T	44	12.2%
4	V726A	NM_000243.2(MEFV): c.2177T > C	44	12.2%
5	M680I	NM_000243.2(MEFV): c.2040G > A or c.2040G > C	35	9.7%
6	M694V	NM_000243.2(MEFV): c.2080 A > G	21	5.8%
7	R761H	NM_000243.2(MEFV): c.2282G > A	6	1.7%
8	P369S	NM_000243.2(MEFV): c.1105 C > T	3	0.8%
9	R202Q	NM_000243.2(MEFV): c.605G > A	3	0.8%
10	I692del	NM_000243.2(MEFV): c.2076_2078del	2	0.6%
11	L110P	NM_000243.2(MEFV): c.329T > C	1	0.3%
	Total		362	100%

### M694V and ethnic distribution

All patients carrying the M694V variant were Egyptian. None had a personal or family history of amyloidosis. The low frequency of M694V and absence of amyloidosis cases support the observation that FMF in the Egyptian population manifests a milder disease form.

### Comparative genotype–phenotype analysis

The clinical characteristics of patients carrying the most prominent homozygous mutation in our study (homozygous E148Q; *n* = 23, 8.3%) were compared with other patients confirmed molecularly as FMF with homozygous mutation, as shown in Table 4. Patients with E148Q homozygous mutation was found to have significantly lower initial serum amyloid children than other patients (*p*-value = 0.001) and lower amyloid A median (*p*-value = 0.030). They mostly responded to a lower dose of colchicine than other patients (*p*-value = 0.028). In other homozygous mutations, significance was not found between age and frequency of mutation. The most common clinical symptoms of the patients were abdominal pain (97.89%) and fever (92.46%). Data were complete for all variables included in Table 4, with no missing values requiring imputation. The ‘homozygous non-E148Q’ group (*n* = 18) comprised a heterogeneous set of variants, including A744S, M680I, M694I, V726A, and M694V, which were analyzed collectively due to the small number of patients in each individual genotype category.

**Table 4:** Comparison of clinical characteristics of patients carrying homozygous E148Q vs. homozygous non-E148Q patients.

Variables	Gene mutation		
	Homozygous E148Q *N* = 23	Homozygous Non-E148Q *N* = 18	*P*-value
Gender			
Male	12 (52.2%)	6 (33.3%)	0.148
Female	11 (47.8%)	12 (66.7%)	
Initial serum amyloid			
Normal	20 (86.9%)	8 (44.4%)	0.001
Raised	3 (13.0%)	10 (55.5%)	
Level of amyloid A (median, IQR)	13.0 (11.7)	50.6 (70.3)	0.030
Number of attacks /year			
< 6	13 (56.5%)	11 (61.1%)	0.804
≥ 6	10 (43.5%)	7 (38.9%)	
Attack duration			
< 72	19 (82.6%)	17 (94.4%)	0.112
≥ 72	4 (17.4%)	1 (5.56%)	
Colchicine dose response			
< 1.5 [mg/day]	18 (78.2%)	8 (44.4%)	0.028
1.5–3[mg/day]	5 (21.7%)	10 (55.5%)	

Four patients were excluded from genotype–phenotype correlation analyses, as no molecular data were available to allow accurate classification. Accordingly, they were not included in Tables 2, 3 and 4, which specifically present analyses stratified by MEFV genotypes.

## Discussion

This study is the largest cohort evaluated for FMF in Egypt to the best of our knowledge. This multicenter study provides a comprehensive overview of the clinical, demographic, and molecular characteristics of FMF in Egyptian patients. Our findings highlight a high prevalence of the E148Q variant, a predominance of mild clinical phenotypes, relatively low frequency of fever attacks, and predominance of female gender, suggesting a less severe disease spectrum compared to neighboring Mediterranean populations.

In our study, the patients’ age was 22.6 ± 12.3 years old. Previous studies have shown that the course of FMF can differ depending on the age at which disease-related symptoms appear. Patients with early onset likely to have more severe symptoms and require larger ultimate colchicine doses^18^. However, patients’ age at the onset of clinical symptoms is highly related to illness severity. Patients over the age of 12 at the first attack had a lower frequency of fever episodes compared to younger patients. Patients less than five years of age at the first attack demonstrated more severe episodes^19^. The elder age of the patients in our cohort goes with the mild symptoms which originate from the prevalence of mild variants as discussed later.

Our study included 173 female (61.8%) and 107 male (38.2%) which means that the female-to-male ratio is 1.6:1 with no statistically significant difference. In agreement with Talaat et al. in the Egyptian Population^20^, Shrateh et al.^21^ and Duşunsel et al.^22^ (in Palestinian and Turkish children; respectively) where female-to-male ratio was found to be high (1.2:1). Other studies reported a male predominance with a female-to-male ratio of 1:1.2 in Turkish populations^23^, or equal prevalence between Arab males and females^24^. In this study, FMF patients in both genders have similar clinical findings, illness severity scores, and treatment compliance, which goes with previous studies^25^, although female patients are more likely to have symptoms including anxiety, sadness, migraines, and headaches, in addition males get diagnosed with FMF early than females^26^.

Although FMF is classified as a fever, fever was documented in only 19.6% of patients at presentation; abdominal pain was the most common presentation, seen in 62.1%, followed by gastrointestinal disorders (30.7%), musculoskeletal involvement (19.3%), chest pain (6.4%) and mucocutaneous involvement (5.4%) (Fig. 1). The clinical presentation of our FMF patients resembled those of Ahmed et al., 2022 which was carried out on Egyptian patients, as well. They found that abdominal pain was among the most common presentation (74%), associated with vomiting (54%). Fever was experienced in only 28% of the cases followed by arthralgia (25%), chest pain and skin rashes (9%)^27^.

The reported incidence of fever in FMF has been estimated as 82.9–93.1%^28^. Cases with afebrile FMF attacks have also been reported^29^. In our cohort, fever was relatively low frequent in 19.6% of enrolled patients. This discrepancy can be attributed to high predominance of the E148Q mutation among participants. E148Q mutation is associated with milder FMF symptoms, due to lack of amyloidosis. Additionally, clinical heterogeneity is observed in patients who are homozygous for E148Q, ranging from absence of symptoms to severe symptoms^30^ explaining this observed discrepancy.

Colchicine remains the cornerstone of FMF management, preventing both attacks and amyloidosis^31^. Our cohort exhibited excellent therapeutic response with moderate dosage. Nearly 46.6% of the patients required < 1.5 mg/day, 47.4% required 1.5–3 mg/day, and only 6% needed > 3 mg/day. This distribution indicates that most patients maintained adequate control with low-to-moderate doses, consistent with a mild clinical phenotype. Our findings were in accordance with Ahmed et al.^27^. Occasional use of higher doses (> 3 mg/day) was limited to adults with frequent attacks or larger body mass indices, not due to colchicine resistance. A more general dose range for the maximum recommended colchicine dose was agreed, with limiters of age, tolerability and signs of toxicity, and the importance of^32^.

In our study, 14 patients (5.1%) had no mutations according to the used test strip despite having typical symptoms. We carried out the genotyping using a teststrip as mentioned before, which carry the most common mutations on the MEFV gene not all the mutations, therefore patients carrying rare variants might show negative results. That’s why we keep those negative patients as they carry typical symptoms. This data might show the efficacy of the teststip in diagnosing patients in our cohort.

The most frequently observed allele in our study was E148Q (*n* = 143, 39.5%), followed by M694I (*n* = 59, 16.3%), A744S and V726A (*n* = 44, 12.2% for each), then M680I (*n* = 35, 9.7%) and M694V (*n* = 21, 5.8%). At least one of these main five founder mutations was present in 240 patients (87%). Thirty-nine patients (14%) were homozygote for one of the main five founder mutations. In our study, the most common homozygous gene mutation was E148Q (*n* = 23, 8.3%), followed by A744S (*n* = 6, 2.2%). Likely, E148Q mutant allele was the most encountered mutation in studied Egyptian patients at Kaf Elsheikh, with a frequency of 31%, followed by M6801 (G/A) 8%^27^.

El-Garf et al. performed similar study on 136 Egyptian patients, the most frequent gene mutations in their studied group were V726A (41.2%), M694V (32.4%), M680I (29.4%), E148Q (25%) and M694I (20.6%). A744S was only found in 4.4% of the patients which comes in the eighth grade after I672del and R761H. The most common homozygous gene mutations were E148Q and M694V (*n* = 12, 8.8% for each), however no patients in their cohort had homozygous A744S^13^. In the same context, El Gezery et al. found that the most common FMF causing mutations in an Egyptian cohort were M694I (34%), E148Q (22.7%), V726A (15.6%), M680I (12.1%), and M694V (7.8%)^33^.

Most recently, Abouzaid et al. calculated the frequency of MEFV variants in FMF affected Egyptians. The most common variant was M694I (29.3%), followed by M680I (20.6%), E148Q (10.3%), M694V (8.6%), and V726A (6.9%)^34^. Compared to previous Egyptian studies, we concluded that the leading FMF-causing mutations in Egypt are V726A, M694I, E148Q, M694V and M680I mutations. However, there were some discrepancies in frequencies; for example, the E148Q mutation, which was the first most prevalent variant in our study (39.5%), was found to be the second most prevalent variant in El Gezery et al. study (22.7%)^33^ and the fourth most prevalent variant in El-Garf et al. study (25%)^13^. A summary of the MEFV mutation frequencies in Egyptian patients in different studies is tabulated in Table 5.

**Table 5:** Comparison of MEFV mutation frequencies in Egyptian Studies.

Study	Most frequent mutations (descending order)	Key notes
Current study	E148Q (39.5%), M694I (16.3%), A744S (12.2%), V726A (12.2%), M680I (9.7%), M694V (5.8%)	E148Q most frequent
El-Garf et al.	V726A (41.2%), M694V (32.4%), M680I (29.4%), E148Q (25%), M694I (20.6%)^13^	A744S only 4.4%; E148Q ranked 4th
El-Gezery et al.	M694I (34%), E148Q (22.7%), V726A (15.6%), M680I (12.1%), M694V (7.8%)^33^	E148Q ranked 2nd
Abouzaid et al.	M694I (29.3%), M680I (20.6%), E148Q (10.3%), M694V (8.6%), V726A (6.9%)^34^	M694I dominant

This somehow goes with other studies conducted on Arab populations which showed the same mutational profile but with different frequencies. M694V was found to be the most common mutation in Palestine (43.4%; followed by E148Q (15.6%), V726A (5.7%), A744S (4.1%), R202Q (4.1%), M694I (3.3%), and M694V + V726A (3.3%))^21^, Lebanon (20.6%; followed by E148Q (17.9%) and V726A (16.0%). R202Q (12.6%), M694I (11.8%), and A744S (10.7%))^35^, Syria (29.7%)^36^ and (45.8% followed by V726A (26%), M694I (13.9%), E148Q (6%) and M680I (4.8%))^37^, Jordan (30% followed by E 148Q 21.5%, V 726 A 20%, M6801 G/C 9%, M6941 8.3%, P369s 3.7%, A744S 3.1%)^38^, Morocco (47%, followed by M694I (32%), A744S (6.5%), M680L (4%), M694del (2%) and E148Q (6.5%))^39^, and Tunisia (27%, M680l (32%), E148Q (18%), M694l (13%), V726A (5%), A744S (3%), R761H and l692DEL (1% for each)^40^. M694V and M694I are the most frequent mutations among FMF Arab patients of North Africa in Algeria (5%, 80%), Tunisia (50%, 25%) and Morocco (49%, 37%), patients; respectively^41^.

In Turkey, M694V was the most frequent mutation (51.4%), followed by M680I (14.4%) and V726A (8.6%)^23^. More recently, the most frequently observed mutation in another Turkish center was R202Q (1319, 19.55%) followed by E148Q (*n* = 476, 7.05%), M694V (*n* = 439, 6.51%), V726A (*n* = 146, 2.16%) and M680I (*n* = 135, 2%). In addition,, a new mutation called S145G (p. Ser145Gly, c.433 A > G) was identified in exon 2 of the MEFV gene in a case clinically diagnosed as FMF^42^. A summary of the MEFV mutation frequencies in Arab and Middle East studies is tabulated in Table 6.

**Table 6:** Mutation frequency trends in other Arab and Mediterranean Populations.

Country	Most frequent mutations reported
Palestine^21^	M694V (43.4%), E148Q (15.6%), V726A (5.7%), A744S (4.1%), R202Q (4.1%), M694I (3.3%)
Lebanon^35^	M694V (20.6%), E148Q (17.9%), V726A (16.0%), R202Q (12.6%), M694I (11.8%), A744S (10.7%)
Syria^36,37^	M694V (29.7–45.8%), V726A (26%), M694I (13.9%), E148Q (6%), M680I (4.8%)
Jordan^38^	M694V (30%), E148Q (21.5%), V726A (20%), M680I (9%), M694I (8.3%), A744S (3.1%)
Morocco^39^	M694V (47%), M694I (32%), A744S (6.5%), E148Q (6.5%), M680L (4%)
Tunisia^40^	M680I (32%), E148Q (18%), M694I (13%), V726A (5%), A744S (3%)
Turkey^23,42^	M694V (51.4%), M680I (14.4%), V726A (8.6%); newer report: R202Q most frequent

The most common clinical symptoms of our patients were abdominal pain (62.1%) and GI disorders (30.7%). Significant increase in abdominal pain and arthritis was found in patients with homozygote M694V mutation compared to those with E148Q mutation. All patients with amyloidosis had M694V gene mutation. The increased frequency of V726A gene mutation and the rarity of amyloidosis in this study suggest that Egyptian patients may have a milder form of FMF compared to other populations. M694V gene mutation may be associated with increased frequency of abdominal pain, arthritis and the presence of amyloidosis^13^. In our cohort, the M694V variant, was detected in only 5.8% of patients. All M694V-positive patients enrolled in this study were Egyptian, and none had a family history of amyloidosis. This finding highlights a significant ethnic variation in FMF severity and suggests a possible protective background in Egyptian carriers or environmental modulation of gene expression. The relative absence of amyloidosis and the favorable response to standard colchicine doses further reinforce this interpretation.

The phenotypic expression of the disease is attributable to the gene mutation, which also contributes to disease severity. Previous reports suggested that M694V show more severe phenotype than other genotypes^43^. Moreover, an association has been shown between the development of amyloidosis (which is the main concern in FMF) and the presence of mutations at position 694 within the MEFV gene^13^. M694V was observed in severe disease and in patients with amyloidosis^33^. The frequency of M694V in Egyptian FMF patients was not consistent among published studies. It was not detected in one study^44^, detected in only 7.8%^33^, and reached up to 32.4% in another study^13^. In our study, M694V is the sixth most frequent allele (*n* = 21, 5.8%), only 2 patients had homozygous M694V, 19 patients had at least one M694V allele (6.9%). Fortunately, M694V is not highly common among Egyptians, this might reveal that FMF in Egyptians is milder than other populations with higher frequency of M694V. This observation was also supported by the fact that most of our cohort (94%) responded to mild to moderate colchicine dose (< 3 mg/day) as mentioned before, which is in accordance with Ahmed et al.^27^. Further clinical research across broader geographical regions is needed to support this view.

The clinical characteristics of patients carrying the most prominent homozygous mutation in our study (homozygous E148Q; *n* = 23, 8.3%) were compared with other patients confirmed molecularly as FMF with homozygous mutation, as shown in Table 4. Patients with E148Q homozygous mutation was found to have significantly lower initial serum amyloid children than other patients (*p*-value = 0.001) and lower amyloid A median (*p*-value = 0.030). They mostly responded to a lower dose of colchicine than other patients (*p*-value = 0.028). These results go with previous studies that revealed that children carrying *E148Q* variants meet the diagnostic criteria of FMF, however, they have milder disease course than children homozygous for another pathogenic *MEFV* variants both clinically and in laboratory data. They have milder clinical features, incomplete attacks, lower CRP, ESR and serum amyloid A^45^. In our cohort, SAA elevation was observed in approximately half of the patients, consistent with subclinical inflammation even in remission phases. This result supports the recommendation that SAA monitoring can aid in long-term risk assessment for amyloidosis, even among clinically stable FMF patients^34,46^. Such differences may reflect distinct genetic backgrounds, environmental influences, and healthcare access patterns. This finding enhances the importance of population-specific registries to improve FMF diagnosis and management.

It was a debate on whether E148Q variant is an insignificant variant, a disease-causing variant with low penetrance and mild symptoms, or a significant variant in specific ethnic groups. Awaad et at., assess the clinical characteristics and severity of FMF in patients homozygous for the E148Q variant and concluded that E148Q variant results in mild to moderate FMF severity, supporting its pathogenic role in particular ethnicity. These results contribute to understanding the clinical significance of the E148Q variant and considering the patient’s need for Colchicine treatment^47^. E148Q has been associated with IgA vasculitis, Ulcerative Colitis and the need for surgery, Crohn’s disease and perianal injury. It has also been shown to increase the risk of developing AA amyloidosis alone or in combination with other auto-inflammatory diseases causing mutations. Moreover, it has been linked to Behçet’s disease, and recurrent aphthous stomatitis, Rheumatoid Arthritis severity, and Multiple Sclerosis suggesting a shared inflammatory pathway involving pyrin–inflammasome regulation^48^.

It is noteworthy that certain variants occur as part of defined haplotypes, as reported in ClinVar. The E148Q variant has been described within two distinct haplotypes: one in combination with L110P (NM_000243.3(MEFV): c.[329T > C;442G > C]) and another with M694I (NM_000243.2(MEFV): c.[442G > C;2082G > A]; OMIM: 608107.0018). In our cohort, L110P was identified only once in a compound heterozygous patient carrying E148Q as well, whereas the E148Q/M694I combination was observed in 13 patients, representing the most frequent compound heterozygous genotype. Similarly, the V726A variant has been reported as part of a genotype with M694V (NM_000243.3(MEFV): c.[2080 A > G]; [2177T > C]). Despite the relatively high frequency of both variants, only one patient in our cohort was identified as compound heterozygous for this combination.

We acknowledge that although E148Q was the most frequently detected variant in our cohort and was associated with a milder clinical phenotype, this finding should be interpreted with caution. We emphasize that the high prevalence of E148Q in our population may partly explain the relatively mild disease expression observed; however, its independent pathogenic role remains debated. Importantly, we have highlighted that clinical diagnosis remains the cornerstone of FMF, particularly in patients carrying a single heterozygous variant such as E148Q. Genetic findings should therefore be interpreted in the context of clinical presentation and established diagnostic criteria, rather than used in isolation.

The association between FMF and some MEFV gene mutations has been clearly established, however, controversy exists regarding the role of R202Q (c.605G > A) located on exon 2, where Q (glutamine) substitutes R (arginine). R202Q genotype was not reported among Egyptians with FMF. This may be due to using different test strips which lack this variant. In our study, it accounts for 0.8% only of the alleles. Among Arabs, it was only documented in few studies in Palestine (4.1%)^21^ and Lebanon (12.6%)^35^. However, the R202Q genotype was reported to be the most prevalent variant in FMF Turkish patients in a study done by Coşkun et al. who found it in 55.8% of his patients^49^ and Gunesacar et al. in 21.35%^50^. R202Q is a polymorphism, it might be a disease-causing mutation associated with some FMF patients. Thus, R202Q polymorphism should be included in routine molecular diagnosis of FMF patients^51,52^. Further research across broader geographical regions is needed to determine the diagnostic significance of this polymorphism in affected patients.

It is worth mentioning that, R202Q polymorphism was noticed in linkage disequilibrium with M694V^53^, which is previously reported in Turkish FMF patients as the most common MEFV mutation^23^. Such that 50% of patients with heterozygous M694V were found having homozygous R202Q polymorphism, as well as 88.8% of patients with homozygous M694V^53^.

## Conclusion

This multicenter study provides comprehensive insights into the clinico-demographic and molecular characteristics of FMF in Egyptian patients. Clinically, abdominal pain was the most common presentation, followed by GI and musculoskeletal manifestations, challenging the classical febrile phenotype.

Genetically, MEFV mutations were detected in 93.6% of cases, with E148Q (39.5%) being the most frequent allele, followed by M694I (16.3%) and V726A/A744S (12.2% each). Notably, homozygous E148Q patients (8.3%) exhibited milder disease, with lower serum amyloid A levels (*p* = 0.001) and better response to colchicine (*p* = 0.028). The predominance of the E148Q variant and the relatively low frequency of M694V indicate a milder clinical phenotype among Egyptian FMF patients.

Therapeutic outcomes showed that half of patients responded to standard colchicine doses (1.5–3 mg/day), with only 6% requiring higher doses. Importantly, no gender-based differences were observed in attack frequency, duration, or treatment response. The low fever prevalence (19.6%), limited proteinuria, and strong response to standard or low-dose colchicine therapy highlight a favorable disease course with minimal progression risk.

Our findings emphasize the importance of integrating both Tel-Hashomer and Eurofever/PRINTO criteria to capture atypical or mild cases in populations where the disease may present with non-febrile manifestations. Further longitudinal studies with extended follow-up are warranted to assess long-term outcomes, amyloidosis risk, and genotype-specific therapeutic response in Egyptian and regional FMF cohorts.

## Study strengths and limitations

Strengths include the large multicenter design, integration of both Tel-Hashomer and Eurofever/PRINTO criteria, and the combined clinical-genetic assessment.

Limitations involve the lack of long-term follow-up data and potential underestimation of amyloidosis due to the relatively young cohort. Future studies should include prospective monitoring for subclinical amyloidosis and genotype-specific therapeutic response.

The findings derived from comparisons with the homozygous non-E148Q group should be interpreted with caution. This comparator group was relatively small and genetically heterogeneous, encompassing multiple MEFV variants with potentially distinct phenotypic effects. Such heterogeneity may have introduced variability and influenced the observed associations, including statistically significant differences in serum amyloid A levels and colchicine dose response. Therefore, these results may not be generalizable to specific non-E148Q genotypes individually. Future studies with larger sample sizes are warranted to enable stratified analyses of individual variants and to better delineate genotype–phenotype correlations.

Parental and extended family testing was not systematically performed in this study due to logistical and resource limitations. However, it is important to note that inzFMF, genetic screening of the *MEFV* gene is recommended for first-degree relatives and other at-risk family members, preferably at an early stage, even in the absence of clinical symptoms. Such screening may facilitate early diagnosis, appropriate monitoring, and timely initiation of management.

The missing data may have influenced the precision of estimated associations and could limit the generalizability of certain findings. Analyses were conducted using available-case (complete-case) methods, and no imputation techniques were applied. Importantly, the primary conclusions of the study—particularly those related to genotype distribution and overall clinical patterns—were based on variables with high data completeness and were not materially affected by the missing data.

## Data Availability

All data generated or analysed during this study are included in this published article.
